# Relationship between the Status of Third Molars and the Occurrence of Dental and Periodontal Lesions in Adjacent Second Molars in the Polish Population: A Radiological Retrospective Observational Study

**DOI:** 10.3390/jcm13010020

**Published:** 2023-12-19

**Authors:** Daniel Poszytek, Bartłomiej Górski

**Affiliations:** Department of Periodontology and Oral Mucosa Diseases, Medical University of Warsaw, 02-097 Warsaw, Poland; bartlomiej.gorski@wum.edu.pl

**Keywords:** alveolar bone loss, dental caries, root resorption, molar, third, tooth, unerupted

## Abstract

The aim of this study was to evaluate the effect of third molars on caries, external root resorption, and alveolar bone loss on the distal surface of adjacent second molars. A total of 2488 panoramic radiographs of adult Poles were evaluated. Third molars were classified, according to eruption status, into non-impacted, partially, or completely impacted, and according to angulation into horizontal, mesioangular, vertical, and distoangular. Completely impacted third molars were assigned as reference group. The odds ratios (ORs) and 95% confidence intervals for the occurrence of the above-mentioned pathologies were 1.39 (1.09–2.21), 6.51 (3.72–10.11), and 2.42 (1.22–4.09), respectively, for second molars with adjacent erupted third molars and 1.54 (1.11–2.82), 10.65 (7.81–20.19), and 5.21 (3.38–10.81), respectively, when partially impacted third molars were next to second molars. The ORs of lesions were significantly higher for horizontally and mesioangularly impacted third molars. Within the limitation of a radiological study, it might be concluded that the presence of erupted third molars is a risk factor for caries, while the presence of impacted third molars increases the risk of root resorption and bone loss on the distal surface of second molars.

## 1. Introduction 

Tooth impaction is a phenomenon in which a tooth does not erupt through the gingival mucosa within the expected timeframe, specific to a given tooth group. [1] Based on radiological examination, teeth can be divided into completely impacted, when the tooth is fully surrounded by bone tissue, and partially impacted, when a fragment of the tooth is located outside the bone but has not reached the occlusal plane [2]. 

Third molars (M3s) are the most frequently impacted teeth in the permanent dentition of humans [3]. M3s make up 98% of all types of impacted teeth [4]. According to data, the prevalence of M3 impaction ranged from 16.7% to 66.86% [5]. A recent meta-analysis indicated worldwide M3 impaction prevalence at 24.40% [6]. The odds of M3 impaction in the mandible were 57.58% higher than in the maxilla (*p* < 0.0001). Neither sex nor population were found to significantly affect the frequency of M3 impaction. Mesioangular impaction was the most common variety (41.17%), followed by vertical (25.55%), distoangular (12.17%), and horizontal (11.06%). However, the prevalence of distoangular and bucco-lingual inclination was significantly higher in the upper I-M3 [7].

The primary reason for the retention of M3s is a deficit of space in the dental arch, resulting from evolutionary changes in the reduction in the bone base of the maxilla and mandible [8]. Less common causes of third molar impaction (I-M3) are incorrect positioning of the tooth bud, intraosseous pathologies, such as cysts or odontogenic tumors, or genetic diseases, such as cleidocranial dysplasia and ectodermal dysplasia [9,10]. The high incidence of third molar impaction makes the management of third molars an important role in public health. The retention of M3s may be asymptomatic or symptoms may occur due to troubles with eruption. Pain, swelling, trismus, and general symptoms such as fever and malaise are common reasons for patients to visit a dental office and may significantly affect their quality of life [11,12]. The literature also mentions long-term complications related to the presence of impacted third molars, which may affect not only the M3 but also surrounding tissues and teeth. Partially impacted M3s were associated with odontogenic infections, such as caries and periodontal diseases [13]. On the other hand, completely impacted M3s were predominantly related to non-inflammatory conditions, such as dentigerous cysts, odontogenic keratocysts, and ameloblastomas [14]. The aforementioned intraosseous pathologies, as well as an incorrect position of the third molar bud, may also contribute to second molar impaction. This rare phenomenon occurs in 0.03% to 0.65% of adolescents [15].

M3s may also be a factor impeding oral hygiene in the posterior areas. A high impaction rate, together with misalignment in all three spatial axes, especially in the mesial direction, causes extremely favorable conditions for the growth of oral cavity bacteria, which ultimately leads to the development of caries in adjacent second molar teeth (M2), and localized alveolar bone loss (ABL). Further development of those diseases may lead to either pulpitis or severe defects of periodontal tissues and increased mobility of M2s, thus carrying the risk of M2 loss. [16] Moreover, due to pressure exerted by eruption forces, there is a risk of external root resorption (ERR) in M2s adjacent to I-M3s, which may reach the root canal and lead to pulp inflammation and, consequently, pulp necrosis [17]. The cervical or apical location of contact points between the teeth, as well as mesioangular, horizontal, and inverted tooth inclination, were all associated with the highest risk of ERR [18,19]. A number of studies examined the relationship between the status of M3s and dental, as well as periodontal, lesions in adjacent M2s; however, to date, no systematic guidelines are available to allow for adequate predictions regarding the development of M3 pathologies, with respect to impaction status, angulation, and the comprehensive clinical picture [20]. To the best of our knowledge, the exact circumstances under which M3s should be extracted remain unclear [21].

On the basis of the available literature, we suspect that the presence of M3s increases the likelihood of caries, external root resorption, and alveolar bone loss occurring on the distal aspect of adjacent second molars. Therefore, the aim of this study was to evaluate this hypothesis based on panoramic radiographs taken among adult Poles. The null hypothesis (H_0_) is that the presence and position of M3s do not raise the chances of any mentioned pathologies in M2s.

## 2. Materials and Methods

### 2.1. Patient Selection

This retrospective observational study was conducted in accordance with the Declaration of Helsinki and approved by the Ethics Committee of the Medical University of Warsaw (MUW, protocol code AKBE/291/2019). Panoramic radiographs of 2488 patients, referred to the Department of Periodontal and Oral Mucosa Diseases at MUW between January 2020 and December 2022, were evaluated retrospectively. The assessment was performed by a single examiner, who is a dental specialist in periodontology (D.P.). The minimum age for inclusion was 19 years since this is when M3s usually begin to erupt and are thus in the vicinity of M2s and might potentially affect them [22]. The upper age was not set.

Panoramic radiographs were taken in the Department of Dental and Maxillofacial Radiology at MUW. All projections were obtained using a Vatech Pax-I device (Vatech, Prague, Czech Republic, 70–85 kVp; 4–10 mA; exposure time of 9–19 s; 100.7 mGy × cm^2^). The photographs were analyzed using a digital viewer (MicroDicom-DICOM viewer, MicroDicom Ltd., Sofia, Bulgaria, microdicom.com (accessed on 18 December 2023). The exclusion criteria applied to the panoramic radiographs are included in Table 1.

**Figure 1:**
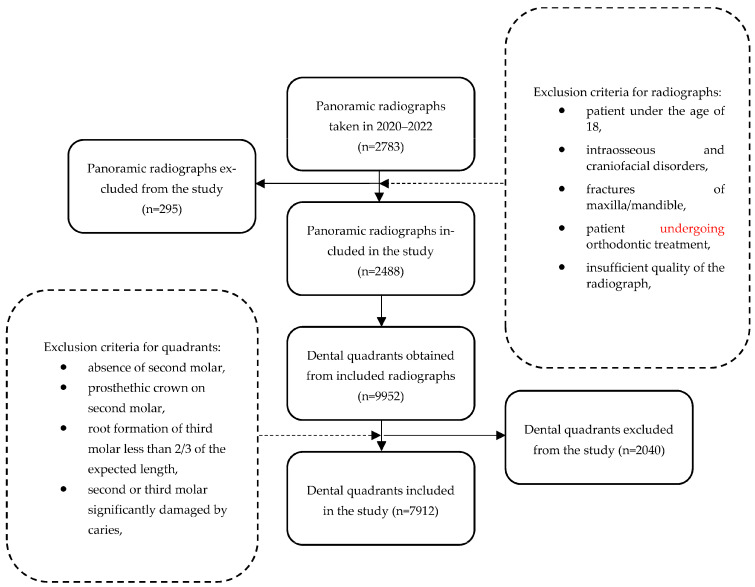
Flowchart of the qualification of dental quadrants.

A total of 2783 panoramic radiographs were retrieved; 295 subjects were excluded on the basis of the exclusion criteria, and 2488 panoramic radiographs were accepted. Ultimately, 7912 dental quadrants were qualified for further evaluation (Figure 1). The age and sex of the patients were recorded based on the data provided in the documentation.

### 2.2. Panoramic Radiograph Analysis

**Figure 2:**
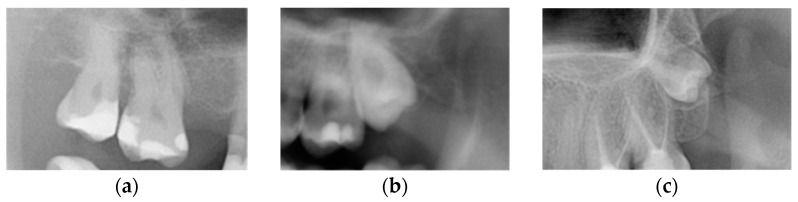
Third molars depending on the eruption status: (**a**) non-impacted (erupted) M3, (**b**) partially impacted M3, and (**c**) completely impacted M3.

M3s were divided, according to the eruption status into (1) non-impacted (N-M3), when the tooth reached the occlusal plane, (2) completely impacted (I-M3), when the tooth was completely surrounded by bone, and (3) partially impacted M3, when the crown of a tooth was situated above the bone edge, but had not reached the occlusal plane (Figure 2) [2].

**Figure 3:**
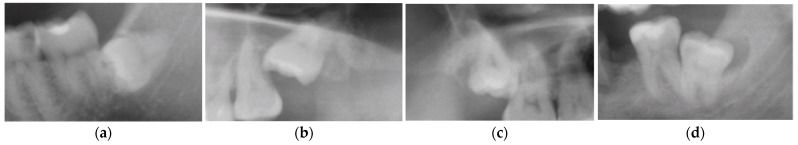
Third molar angulation: (**a**) horizontal, (**b**) mesioangular, (**c)** vertical, and (**d**) distoangular.

Additionally, M3s were categorized based on Winter’s classification in terms of their position in the dental arch relative to the long axis of M2s, according to the following criteria: (1) horizontal (angle between the long axes of an M2 and an M3 between 80° and 100°), (2) mesioangular (from 10° to 80°), (3) vertical (from −10° to 10°), and (4) distoangular (from −10° to −80°) (Figure 3) [23].

**Figure 4:**
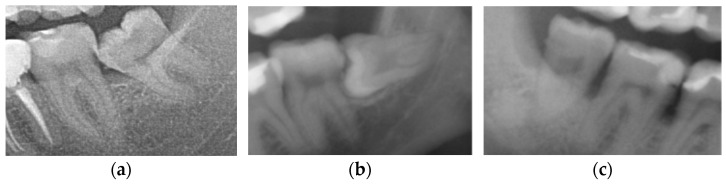
Radiologic view of pathologies in second molars and adjacent tissues: (**a**) caries on the distal surface of M2s, (**b**) external root resorption of the distal root of M2s, (**c**) bone loss on the distal aspect of M2s.

M2s and surrounding tissues were then analyzed for the presence of the following pathologies based on the guidelines by Al Khateeb et al. [24]: (1) caries on the distal surface of the tooth, described as a radiographically clear lesion with no direct contact with the crown of an M3, (2) external root resorption (ERR) on the distal surface of the root, defined as loss of tooth substance, caused by direct contact between M2s and the crown of an M3, (3) alveolar bone loss (ABL) of the alveolar process of the maxilla, or the alveolar part of the mandible distal to M2s, greater than 20% of the length of the distal root (Figure 4).

Only the occurrence of the above-mentioned diseases was assessed, not their advancement.

### 2.3. Statistical Analysis

Statistical analysis was conducted using Statistica 13.3 (Dell Technologies Inc., Round Rock, TX, USA (2016); Dell Statistica (data analysis software system), version 13.3; dell.com (accessed on 18 December 2023). Data are presented as frequencies and ratios. To evaluate the impact of M3 status on M2 pathologies, a multivariate logistic regression analysis was used. The odds ratio (OR) with 95% confidence intervals (CIs) was calculated separately for caries, ERR, and ABL. The presence of the analyzed pathologies was compared between M3s that were absent, partially impacted, and completely impacted. Moreover, the OR of the above-mentioned pathologies was assessed in relation to M3 angulation (horizontal, mesioangular, vertical, and distoangular), as well as patient age and sex. Quadrants with completely impacted M3s were used as reference groups for ORs. In the data set for logistic regression, the absence of multicollinearity was detected using the variance inflation factor method. The level of significance was set at 0.05.

## 3. Results

### 3.1. Demographic and Radiographic Findings

A total of 2488 X-rays were included in this study (1055 men and 1433 women; aged ≥ 19 years, mean age: 42.2 years). The exclusion criteria were applied to the quadrants, and 7912 quadrants qualified for this study. Among all 2488 subjects, 1842 participants (74.04%) had at least one N-M3, 631 participants (25.23%) had at least one partially impacted M3, and 303 participants (12.18%) had at least one completely impacted M3. With respect to the second molars, 1738 of which showed signs of distal caries, 141 had external resorption of the distal root and 1571 were diagnosed with bone loss on the distal aspect of the tooth (Table 2, Table 3 and Table 4).

The number of M3s, according to their angulation, is presented in Table 5. Vertically positioned M3s were the most prevalent in every category, followed by mesioangular M3s. Distoangular and horizontal M3s were observed in much smaller numbers.

### 3.2. Multivariate Logistic Regression Analysis

The results of the analysis considering pathologies in M2s in comparison with erupted M3s are presented in Table 6. Completely impacted M3s were assigned as reference a group. All pathologies were more prevalent in the presence of both partially impacted and erupted M3s, although the ORs of carious lesions in M2s adjacent to erupted M3s in the mandible and in the maxilla and mandible altogether were not statistically significant.

Table 7 presents the ORs for caries, ERR, and ABL with respect to the angulation of M3s relative to the long axis of adjacent M2s. The risk of occurrence of every above-mentioned pathology was higher when M3s were placed either horizontally or mesioangularly. Moreover, vertically positioned M3s showed higher ORs for ERR in comparison with the ORs when an M3 was absent.

The ORs for the pathologies, taking into consideration patient age and sex, are presented in Table 8. The ORs for caries and ABL on the distal aspect of M2s increased with patient age, although the ORs for ERR were not statistically significant. Moreover, it was assessed that the likelihood of ABL was higher in male patients, whereas the risk of caries was greater in female patients.

## 4. Discussion

The aim of this study was to evaluate whether the presence of M3s poses a risk of caries, external root resorption, and alveolar bone loss on the distal aspect of adjacent M2s. Based on the data obtained from panoramic radiographs, it was found that the presence of N-M3s was associated with a higher incidence of caries, while the presence of I-M3s increased the incidence of ERR and ABL on the distal surface of M2s. The ORs of dental and periodontal lesions were significantly higher for horizontal and mesioangular I-M3s. The results of our study may assist in predicting which M3s are especially likely to cause the development of the aforementioned pathologies in M2s. This, in turn, may raise awareness among dental professionals and encourage the enrollment of patients in periodic clinical and radiological prophylactic protocols.

Our findings are consistent with prior research on caries in M2s associated with retained M3s. It was implied that plaque may be undisturbed in the approximal area, which is often inaccessible to cleaning devices. Subsequently, the presence of M3s may lead to the appearance of caries on the distal surface of M2s [25]. The prevalence of caries on the distal surface of mandibular M2s, associated with a semi-erupted M3, ranged from 7% to 32% [26,27,28,29,30,31,32]. A large longitudinal study with a 25-year follow-up reported a relative risk ratio (RR) of 2.53, which meant that patients with erupted M3s were at 153% greater risk of caries on the distal surface of M2s in comparison with subjects missing M3s [33]. Soft tissue I-M3s had a caries RR of 0.83, whereas bony I-M3s had a RR of 1.44 in comparison with patients with absent M3s. However, the sample of the above-mentioned study was composed exclusively of male patients. European studies suggested that M2 caries may be present in approximately one in four referrals for the assessment of M3s [34]. A recent meta-analysis confirmed that the presence of mandibular M3s increased the incidence of caries on the distal surface of M2s [35]. Moreover, the risk was higher when M3s were completely erupted (class A—Pell and Gregory), rather than non-erupted (class C—Pell and Gregory) (OR 3.45) when the horizontal position was compared with the vertical (OR 9.12) and distoangular (OR 9.75) positions and when the mesioangular position was compared with the vertical (OR 7.25) and distoangular (OR 9.54) positions. The overall pooled frequency of caries was 23% among patients [34]. A subgroup analysis yielded a prevalence of 36% for mesioangular I-M3s and 22% for horizontal I-M3s. As horizontal and mesioangular M3s exhibited a greater cement–enamel distance from M2s, food retention in the region also increased, leading to cleaning issues and a higher incidence of caries on the distal surface of M2s. It was also implied that the longer the M3s were exposed in the mouth, the greater the chance of caries occurring on the distal surface of M2s [36]. Similar to earlier studies, the present study found that horizontal and mesioangular M3s were most often correlated with the presence of caries on M2s. Given the above, caries on the distal surface of M2s is an associated long-term consequence of M3 retention. Thus, numerous authors recommended the prophylactic removal of semi-erupted, horizontal, and mesioangular M3s in order to prevent the appearance of caries on the distal surface of M2s [37,38].

ERR was associated with mechanical or inflammatory factors such as dental trauma, chronic periodontitis, pressure resulting from orthodontic appliances, cysts, benign or malignant tumors, and close proximity to an unerupted tooth [39]. Previous studies analyzing the ERR of M2s due to M3s, which used panoramic imaging or apical radiographs, reported prevalence varying between 0.3% and 24.2% [24,28,40,41,42,43]. In the present study, the prevalence of ERR was 2.28% when M3s were present and increased to 6.62% in case of partially impacted M3s. However, a prevalence rate as high as 49.43% was reported in previous studies that used CBCT [27,44]. Interestingly, a Chinese study carried out with the use of CBCT reported a lower incidence of ERR (20.17%), including only M3s that were mesially and horizontally impacted [45]. It must be highlighted that image acquisition parameters, such as the voxel size, influenced the detection of ERR, which may help explain some of the discrepancies [46]. In a study by Suter et al. [18], ERR was identified in 31.9% of M2s and was slight in 30.2%, moderate in 1.4%, and severe in 0.3% of the cases. The presence of ERR was significantly associated with direct contact between M2s and M3s, the angle between M2s and M3s with a mean angle measurement of 57.10°, the inclination of M3s, and the location of contact. Mesioangular, horizontal, and inverted impaction of M3s were correlated with increased ERR on the adjacent M2s. The risk percentage for the incidence of ERR was 62.5% for inverted inclination, 54.2% for horizontal inclination, and 47.2% for mesioangular inclination. Moreover, a male predilection was found with a risk percentage of 41.4%. In contrast, no significant sex predilection was found in other studies, as well as in our study. In a recent systematic review and meta-analysis, ERR in M2s was significantly associated with contact with I-M3s and the inclination of M3s [39]. The presence of ERR in mandibular M2s was higher (38.3%) than in the upper arch (33.8%). Taking into account the inclination of I-M3s, the incidence of ERR was higher with transverse, horizontal, and mesio-angular impacted M3s, with 54.5%, 47.5%, and 44.5% occurrence, respectively. M2s in close proximity to mesio-angular I-M3s showed a 50% higher risk of ERR than with vertical I-M3s (RR 0.50). Similar to our study, the majority of the research studies did not find an association between the incidence of ERR and age.

Increased levels of periodontal microbiota in M3 regions were observed, even with very limited clinical symptoms of periodontal issues [47]. The M3 pericoronal region may provide a favored niche for periodontal pathogens in otherwise healthy oral cavities. Another study reported an increased plaque index and bleeding on probing (BOP) around adjacent M2s [48]. The presence of N-M3s was indicated as a potential risk factor for the development of periodontitis in adjacent M2s [27,28,32,49]. N-M3s were correlated with a probing pocket depth (PPD) of at least 5 mm (OR 6.7) and BOP (OR 4.0). What is more, the periodontal status of adjacent M2s was affected by age [50]. Our study indicated that periodontal risk is associated with the presence of lower I-M3s, with higher odds for horizontal and mesioangular positions. The OR was higher in males and increased with age. A recent systematic review and meta-analysis by Yang et al. [51] found that the presence of M3s, especially mandibular N-M3s, negatively affected the periodontal status of adjacent M2s. The prevalence of ABL was 32% in the mandible when compared to 19% of M3s in general. It was higher with N-M3s (25%) than with I-M3s (19%). The higher prevalence of N-M3s might be associated with significant differences in periodontal pathologies between different impaction types. All in all, 19% of M2s showed distal early periodontal defects with the presence of M3s. Moreover, the pooled prevalence for deep periodontal pockets around M2s was 52%. Subgroup analyses showed the prevalence was higher in the mandible (62%) than in the maxilla (43%). In the case of N-M3s, the prevalence of deep periodontal pockets reached 50%. A wide range of studies observed that the removal of M3s, irrespective of being N-M3s or I-M3s, contributed to improvements in the periodontal status of adjacent M2s [48,52,53,54]. Baseline deep PPD, as well as advanced age, led to an unfavorable prognosis. A recent systematic review and meta-analysis reported that lower M3 surgery resulted in a moderate reduction in PPD, clinical attachment level (CAL), and alveolar bone defect (ABD) [55]. At 6 months, the PPD reduction was 1.06 mm, and the remaining PPD was 3.81 mm. Based on only four studies, PPD at 12 months was 4.79 mm, meaning that the periodontal pocket did not resolve completely after surgery. Baseline PPD was strongly associated with the remaining PPD at 6 months.

It is well-known that even asymptomatic M3s frequently become diseased with increased patient age and retention time. Caries and periodontal pathologies were most frequently reported, especially in partially erupted M3s and mesially inclined mandibular M3s [19,27]. Therefore, a number of authors recommend prophylactic removal of M3s since they are often nonfunctional and strategically non-important teeth. The advantages of such management are numerous. First of all, the earlier an M3 is extracted, the lower the odds for the development of caries, ERR, and ABL, as well as pericoronitis, odontogenic cysts, and tumors in that anatomical area. Interestingly, a recent study suggested that radiographic appearance may not be a reliable indicator of the absence of disease within the M3 dental follicle; hence, attention should be paid to peri-coronal radiolucency of follicles less than 2.5 mm in size [56]. Furthermore, there is a decreased likelihood of the occurrence of complications associated with M3 eruption, such as pain, swelling, purulent discharge, or trismus, which may increase the difficulty of extraction, as well as jeopardize postoperative healing. Moreover, as M3 roots may be underdeveloped, root separation may not be required, which makes the extraction easier and less time-consuming, and carries less risk of lingual nerve damage. On the other hand, some believe that there is little evidence for the benefits of M3 removal and there is a higher chance of postoperative complications than the risk of pathologies related to I-M3s. The occurrence of those complications might depend on a patient’s general health and lifestyle, the dental surgeon’s experience, and the anatomy of M3s and surrounding tissues. Apart from complications typical for tooth extraction, such as alveolitis, either sicca or purulent [57], facial swelling [58], or bleeding [59], there are those highly specific for third molar surgery. Postoperative trismus results from inflammatory infiltration of the masticatory muscles, especially the medial pterygoid muscle. It may significantly limit mouth opening and food intake. [60] Inferior alveolar nerve complications are specific to lower M3 surgery and may result either from edema pressing on the nerve or trauma caused by the tooth extraction instruments. In the first case, hypoesthesia lasts a few days as the swelling subsides, while in the second case, regaining the sensory function may take up to several months. Complete loss of sensation is very rare. As the innervation of the inferior alveolar nerve extends to the lower lip, eating and speaking may be problematic. Involuntary biting of the lip is also common, resulting in deep, long-healing traumatic ulcers [61]. Oroantral connection, on the other hand, might happen after upper M3 extraction due to a lack of or extremely thin alveolar bone proper in the apical part of the alveolus. Although radiographs, especially CBCT, might be useful to predict oroantral connection, the Valsalva maneuver or assessment using alveolar curette are far more reliable methods of detecting oroantral communication. After the closure of the communication, the patients must not sneeze or cough with their mouths closed. Airplane flights and diving are also prohibited to ensure the primary healing of the wound [62]. The above-mentioned issues may have a minor or considerable influence on a patient’s quality of life. It is, therefore, of vital importance to use a scientific evidence-based approach in order to establish which variables increase the prevalence of certain pathologies associated with M3s, hence justifying their prophylactic removal. Understanding the risk factors of M3 impaction will aid in determining which clinical policy is optimal for a given patient. It is also important to assess the difficulty of M3 extraction based on, among others, M3 anatomy, alignment, and surrounding structures. For example, the relation between the M3 bud and the mandibular canal must be taken into account, especially if germectomy is considered. Numerous tools are available for these situations, which may help clinicians balance out indications and risk factors [63]. Although a plethora of clinical studies on this topic have been carried out, conflicting results were often observed. This might explain, at least partially, the lack of consensus among dental practitioners. The results of our study suggest that the retention of M3s, especially when impacted in horizontal or mesioangular positions over a long period of time, may constitute a risk factor for caries and ABL and is likely to result in harm to M2s. In this aspect, our observations are in line with other reports and enforce the evidence already present in the literature [13,18,32,55]. As PPD is more likely to increase with age, M3 surgery ought to be carried out before severe periodontitis occurs in order to avoid compromising periodontal defects and significant ABL [32,55]. Moreover, the horizontal or mesioangular position of I-M3s, and the direct contact between M2s and M3s, represent a further risk for ERR [18,27].

To the best of the authors’ knowledge, this is the largest study of this kind that was carried out in Central Europe and in Poland. A total of 2488 panoramic radiographs and 7912 quadrants were positively qualified and meticulously evaluated. Ratios, frequencies, and ORs of caries, ERR, and ABL on the distal surface of the adjacent M2s were depicted. On the other hand, the results of this study have some feasible limitations and should be interpreted with caution. First of all, the examination was performed only on the basis of panoramic radiographs without clinical evaluation. The disease outcomes should ideally have been assessed both clinically and radiographically. Visual characteristics of caries and periodontal disease, such as changes in the color and opacity of dental tissues, redness and swelling of surrounding soft tissues, as well as periodontal indices, such as probing pocket depth (PPD), clinical attachment level (CAL), and bleeding on probing (BOP), are necessary to ensure a correct diagnosis. Furthermore, additional factors influencing the incidence of caries, ERR, and ABL in M2s ought to be considered and analyzed. Oral hygiene, recorded in the form of a suitable index, such as plaque index or approximal plaque index, plaque retention factors, such as tooth malposition in the buccolingual direction, or soft tissue defects, as well as saliva deficiency, could all have significantly impacted the presence of the above-mentioned pathologies. Furthermore, all included patients were referred to the Department of Periodontal and Oral Mucosal Diseases at MUW, which may have introduced bias, as these patients could potentially exhibit characteristics different from the general population, such as greater prevalence of periodontal disease—this had the potential to jeopardize the diagnosis of periodontal defects in the M3 area, as they may have originated due to generalized periodontitis. All in all, the presented results may not be applicable to all populations. What is more, the use of panoramic radiographs carried the risk of not detecting all the examined pathologies correctly. A minimum 30% loss of mineral substance, such as tooth or bone, must occur to be visible on a conventional radiograph, such as a panoramic X-ray [64]. Additionally, due to the two-dimensional nature of the projection, there was a risk of overlapping the image with surrounding healthy tissues, resulting in a pathology being unnoticed. This underestimation of the prevalence of caries and ERR could constitute one of the most significant limitations of the present study. As previously mentioned, panoramic radiographs were considered a valid, although less precise method to accurately assess the pathologies in question. Numerous studies confirmed that the best method of diagnosing caries or ERR in posterior teeth is the use of periapical inter-proximal radiographs and CBCT [65]. The greatest advantage of CBCT is the possibility to evaluate the root surface of an M2 in all three planes. On the other hand, periapical radiographs are more reliable in assessing periodontal tissues, and bitewing projections help to assess carious lesions more accurately using a much lower radiation dose. Nevertheless, the decision to use panoramic radiographs was made due to their wide availability, widespread use in general dental practice, and relatively low cost. It is also important to note that the state of M3s was not taken into account in our study. Pathologies in M3s, especially carious lesions, might have had an impact on M2s and their surrounding tissues. While significantly damaged M3s were not included in our study, less developed carious lesions might have contributed to M2 status and hindered a proper diagnosis.

Further longitudinal studies are required in a variety of populations, with well-described clinical and socio-demographic characteristics of the population, to better determine the incidence and the factors that increase the risk of caries, ERR, and ABL on the distal surface of the adjacent M2 with respect to M3 eruption status and angulation. Further studies ought to be conducted to explore this topic in a thorough manner, including the examination of participants’ medical history, such as general diseases, medicaments, diet, and access to dental care, as well as clinical examinations including visual assessment of caries, probing pocket depth, clinical attachment level and oral hygiene indices, supported by radiographic imaging, such as CBCT.

## 5. Conclusions

Within the limitations of this radiological study, it may be concluded that:

- The presence of M3s might increase the incidence of caries, ERR, and ABL on the distal surface of M2s, especially in the long term;
- Caries were more commonly associated with N-M3s, whereas the presence of I-M3s was most likely to increase the risk of ERR and ABL;
- Mesioangular and horizontal positions of I-M3s were significantly more likely to cause caries, ERR, and ABL on the distal surface of M2s.

As the current study is cross-sectional and retrospective in nature, the absence of clinical status evaluations may be considered a serious limitation. On the other hand, the careful selection of patients with detailed exclusion criteria may be regarded as an advantage.

## Figures and Tables

**Table 1:** Exclusion criteria for panoramic radiographs and dental quadrants.

Exclusion Criteria for Radiograph/Quadrant	Justification
Age < 18 years old	Eruption process of M3s usually starts later
Intraosseous and craniofacial disorders	Risk of misdiagnosing pathologies
Craniofacial trauma (e.g., fractures)	Risk of misdiagnosing pathologies
Patient undergoing orthodontic treatment	Risk of misdiagnosing pathologies
Artifacts or insufficient quality of a radiograph	Risk of misdiagnosing pathologies
M3 root formation below two-thirds of the expected length	Large potential of M3s to erupt in a proper position
Quadrant without an M2	No possibility to assess pathologies in M2s
Quadrant with an M2 being restored with a prosthetic crown	No possibility to assess which aspect of an M2 was damaged by caries
Quadrant with an M2 or M3 significantly damaged by caries	No possibility to assess the pathologies in an M2 or the influence of an M3

**Table 2:** The presence of caries, root resorption, and bone loss in second molars depending on the presence or absence of a third molar.

	Caries		Root Resorption		Bone Loss	
	*n*	%	*n*	%	*n*	%
None	655	25.21	20	0.77	448	17.24
Maxilla	419	28.22	16	1.08	335	22.56
Mandible	236	21.20	4	0.36	113	10.15
Present	1083	20.38	121	2.28	1123	21.13
Maxilla	542	20.70	74	2.83	632	24.14
Mandible	541	20.07	47	1.74	491	18.21

**Table 3:** The presence of caries, root resorption, and bone loss in second molars depending on impaction or eruption of a third molar.

	Caries		Root Resorption		Bone Loss	
	*n*	%	*n*	%	*n*	%
Impacted	133	8.72	89	5.83	464	30.41
Maxilla	54	7.84	51	7.40	199	28.89
Mandible	79	9.44	38	4.54	265	31.66
Erupted	950	25.08	32	0.84	659	17.40
Maxilla	488	25.30	23	1.19	433	22.45
Mandible	462	24.85	9	0.48	226	12.16

**Table 4:** The presence of caries, root resorption, and bone loss in second molars depending on the degree of impaction of a third molar.

	Caries		Root Resorption		Bone Loss	
	*n*	%	*n*	%	*n*	%
Partially impacted	109	9.88	73	6.62	414	37.53
Maxilla	43	9.70	44	9.93	184	41.53
Mandible	66	10.00	29	4.39	230	34.85
Completely impacted	24	5.67	16	3.78	50	11.82
Maxilla	11	4.47	7	2.85	15	6.10
Mandible	13	7.34	9	5.08	35	19.77

**Table 5:** The number of third molars with respect to their position in the dental arch, relative to the long axis of a second molar.

	Erupted M3		Partially Impacted M3		Completely Impacted M3	
	*n*	%	*n*	%	*n*	%
Horizontal	6	0.16	114	10.33	48	11.35
Mesioangular	930	24.56	323	29.28	145	34.28
Vertical	2602	68.69	546	49.50	159	37.59
Distoangular	250	6.60	120	10.88	71	16.78

**Table 6:** Odds ratios for caries, root resorption, and bone loss in second molars depending on the degree of impaction of a third molar.

M3 Status	Caries		Root Resorption		Bone Loss	
	OR (95% CI)	*p*-Value	OR (95% CI)	*p*-Value	OR (95% CI)	*p*-Value
Maxilla						
Completely impacted	Reference		Reference		Reference	
Partially impacted	1.44 (1.14–3.11)	<0.0001	9.43 (5.31–12.32)	<0.0001	5.32 (3.11–8.91)	<0.0001
Erupted	1.67 (1.09–2.11)	<0.0001	6.22 (2.18–9.07)	<0.0001	1.34 (1.11–2.20)	<0.0001
Mandible						
Completely impacted	Reference		Reference		Reference	
Partially impacted	1.32 (1.11–2.09)	<0.0001	4.36 (2.82–8.91)	<0.0001	4.94 (3.61–9.49)	<0.0001
Erupted	1.45 (1.09–1.65)	0.02	6.81 (3.92–10.61)	<0.0001	3.45 (1.90–8.93)	<0.0001
Maxilla + mandible						
Completely impacted	Reference		Reference		Reference	
Partially impacted	1.54 (1.11–2.82)	<0.0001	10.65 (7.81–20.19)	<0.0001	5.21 (3.38–10.81)	<0.0001
Erupted	1.39 (1.09–2.21)	0.02	6.51 (3.72–10.11)	<0.0001	2.42 (1.22–4.09)	<0.0001

**Table 7:** Odds ratios for caries, root resorption, and bone loss in second molars depending on the angulation of a third molar.

M3 Status	M3 Angulation	Caries		Root Resorption		Bone Loss	
		OR (95% CI)	*p*-Value	OR (95% CI)	*p*-Value	OR (95% CI)	*p*-Value
Maxilla							
None		Reference		Reference		Reference	
Present							
	Horizontal	1.23 (1.13–1.67)	0.0021	12.31 (7.32–23.90)	<0.0001	1.28 (1.13–3.31)	0.0031
	Mesioangular	1.34 (1.21 1.74)	0.0145	3.65 (2.59–4.04)	<0.0001	1.88 (1.50–2.08)	<0.0001
	Vertical	0.76 (0.43–1.12)	0.0892	1.82 (1.21–2.45)	<0.0001	0.83 (0.61–1.25)	0.6371
	Distoangular	0.26 (0.14–0.47)	<0.0001	0.59 (0.22–0.94)	<0.0001	0.89 (0.54–1.35)	0.7311
Mandible							
None		Reference		Reference		Reference	
Present							
	Horizontal	1.32 (0.62–1.78)	0.0036	17.02 (3.59–23.78)	<0.0001	1.30 (1.13–1.49)	<0.0001
	Mesioangular	1.49 (1.14–1.97)	0.0097	4.16 (2.04–7.56)	<0.0001	1.26 (1.04–1.53)	<0.0001
	Vertical	0.71 (0.51–0.96)	0.0309	1.67 (1.16–2.69)	<0.0001	1.05 (0.79–2.22)	0.2441
	Distoangular	0.57 (0.12–0.84)	<0.0001	0.48 (0.23–0.71)	<0.0001	0.37 (0.12–1.86)	0.7727
Maxilla + mandible							
None		Reference		Reference		Reference	
Present							
	Horizontal	1.68 (1.43–2.13)	<0.0001	10.09 (5.58–19.12)	<0.0001	1.67 (1.31–2.17)	<0.0001
	Mesioangular	1.18 (1.05–1.76)	<0.0001	5.17 (2.86–9.15)	<0.0001	1.73 (1.16–2.56)	<0.0001
	Vertical	0.77 (0.67–0.89)	<0.0001	1.55 (1.44–1.42)	0.0029	0.81 (0.24–1.16)	0.3317
	Distoangular	0.34 (0.19–0.56)	<0.0001	0.40 (0.08–1.08)	0.6721	0.91 (0.68–1.84)	0.3711

**Table 8:** Odds ratios for caries, external root resorption, and bone loss in relation to patient age and sex.

		Caries		Root Resorption		Bone Loss	
		OR (95% CI)	*p*-Value	OR (95% CI)	*p*-Value	OR (95% CI)	*p*-Value
Age							
	Maxilla	1.12 (1.04–1.45)	<0.0001	1.03 (0.99–1.11)	0.04353	1.11 (1.08–1.56)	<0.0001
	Mandible	1.03 (0.99–1.13)	0.04882	0.78 (0.45–1.31)	0.328829	1.03 (1.01–1.12)	<0.0001
	Maxilla + mandible	1.05 (1.02–1.13)	<0.0001	0.98 (0.94–1.11)	0.0872	1.04 (1.02–1.07)	<0.0001
Female		Reference		Reference		Reference	
Male		0.79 (0.69–0.89)	<0.0001	0.89 (0.61–1.28)	0.5140	1.16 (1.02–1.35)	<0.0001

## Data Availability

The data presented in this study are available upon request from the corresponding author.

## Abbreviations

ABL	alveolar bone loss
BOP	bleeding on probing
CAL	clinical attachment level
CBCT	cone beam computed tomography
CI	confidence interval
ERR	external root resorption
I-M3	impacted third molar
M2	second molar
M3	third molar
N-M3	non-impacted third molar
OR	odds ratio
PPD	probing pocket depth
RR	risk ratio
